# Characteristics of Low Band Gap Copolymers Containing Anthracene-Benzothiadiazole Dicarboxylic Imide: Synthesis, Optical, Electrochemical, Thermal and Structural Studies

**DOI:** 10.3390/polym13010062

**Published:** 2020-12-25

**Authors:** Ary R. Murad, Ahmed Iraqi, Shujahadeen B. Aziz, Mohammed S. Almeataq, Sozan N. Abdullah, Mohamad A. Brza

**Affiliations:** 1 Department of Pharmaceutical Chemistry, College of Medical and Applied Sciences, Charmo University, Chamchamal 46023, Iraq; ary.murad@charmouniversity.org; 2 Department of Chemistry, University of Sheffield, Sheffield S3 7HF, UK; a.iraqi@sheffield.ac.uk; 3 Hameed Majid Advanced Polymeric Materials Research Lab., Department of Physics, College of Science, University of Sulaimani, Sulaimani 46001, Iraq; 4 Department of Civil Engineering, College of Engineering, Komar University of Science and Technology, Sulaimani 46001, Iraq; 5 King Abdulaziz City for Science and Technology, Riyadh 11451, Saudi Arabia; mmeataq@kacst.edu.sa; 6 Department of Chemistry, College of Science, University of Sulaimani, Sulaimani 46001, Iraq; sozan.abdulla@univsul.edu.iq; 7 Department of Manufacturing and Materials Engineering, Faculty of Engineering, International Islamic University of Malaysia, Kuala Lumpur 53100, Malaysia; mohamad.brza@gmail.com

**Keywords:** conjugated copolymers, optical study, electrochemical properties, thermal properties, powder X-ray diffraction (XRD) analysis

## Abstract

Two novel low band gap donor–acceptor (D–A) copolymers, poly[9,10-bis(4-(dodecyloxy)phenyl)-2,6-anthracene-alt-5,5-(4′,7′-bis(2-thienyl)-2′,1′,3′-benzothiadiazole-*N*-5,6-(3,7-dimethyloctyl)dicarboxylic imide)] (PPADTBTDI-DMO) and poly[9,10-bis(4-(dodecyloxy)phenyl)-2,6-anthracene-alt-5,5-(4′,7′-bis(2-thienyl)-2′,1′,3′-benzothiadiazole-5,6-*N*-octyl-dicarboxylic imide)] (PPADTBTDI-8) were synthesized in the present work by copolymerising the bis-boronate ester of 9,10-phenylsubstituted anthracene flanked by thienyl groups as electron–donor units with benzothiadiazole dicarboxylic imide (BTDI) as electron–acceptor units. Both polymers were synthesized in good yields via Suzuki polymerisation. Two different solubilizing alkyl chains were anchored to the BTDI units in order to investigate the impact upon their solubilities, molecular weights, optical and electrochemical properties, structural properties and thermal stability of the resulting polymers. Both polymers have comparable molecular weights and have a low optical band gap (E_g_) of 1.66 eV. The polymers have low-lying highest occupied molecular orbital (HOMO) levels of about −5.5 eV as well as the similar lowest unoccupied molecular orbital (LUMO) energy levels of −3.56 eV. Thermogravimetric analyses (TGA) of PPADTBTDI-DMO and PPADTBTDI-8 did not prove instability with decomposition temperatures at 354 and 313 °C, respectively. Powder X-ray diffraction (XRD) studies have shown that both polymers have an amorphous nature in the solid state, which could be used as electrolytes in optoelectronic devices.

## 1. Introduction

Organic solar cells are a promising alternative to traditional inorganic semiconducting materials [1,2]. Efforts have been made to design new materials for application in optoelectronic devices such as field effect transistors (FETs) [3,4], light-emitting diodes [5,6], and photovoltaic cells [7]. Research into the development of novel compounds-based anthracene started in 1963 when the Pope and co-workers investigated electroluminescence in anthracene crystals [8]. Anthracene and its derivatives along with the other acenes like tetracene [9] and pentacene [10] have been extensively studied in organic FETs [11]. These materials have good hole mobilities owing to the strong intermolecular interactions in their planar and rigid structures [12].

Anthracene is one of the interesting building blocks as an electron donor unit to construct the medium band gap D–A copolymers for photovoltaic applications [13]. Anthracene has a weak electron-donating ability, therefore anthracene-based D–A copolymers are expected to have low-lying HOMO levels, which are beneficial to obtain high open-circuit voltage (*V*_oc_) values in bulk heterojunction polymer solar cells (BHJ PSCs) [14]. In addition, anthracene could be incorporated into the conjugated polymer chains through its 2,6-positions or 9,10-positions [15,16]. Polymerizing anthracene through its 2,6-positions provides the 9,10-positions for attaching different solubilizing side chains which result in a better processability of the polymers [13]. Anthracene and its derivatives have been widely applied in polymer light-emitting diodes (PLEDs) [17] and FETs [18], while few reports have been published for anthracene in PSCs [19].

Sonar et al. synthesized a D–A copolymer poly[3,6-difuran-2-yl-2,5-di(2-octyldodecyl)-pyrrolo[3,4-*c*]pyrrole-1,4-dione-*alt*-anthracene] (PDPP-FAF) by copolymerizing diketopyrrolopyrrole (DPP) with 2,6-linked anthracene and two furan units as spacers between donor and acceptor units. BHJ PSCs based on PDPP-FAF: PC_71_BM gave a power conversion (PC) efficiency of 2.5% [20].

Iraqi and co-workers developed three alternating copolymers, poly(9,10-bis(4-(dodecyloxy)phenyl)-anthracene-2,6-diyl-alt-(4,7-dithiophen-2-yl)-2′,1′,3′-benzothiadiazole-5,5-diyl] (PPATBT), poly(9,10-bis(4-(dodecyloxy)phenyl)-anthracene-2,6-diyl-alt-(5,6-bis(octyloxy)-4,7-di(thiophen-2-yl)benzo[c][1,2,5]thiadiazole-5,5-diyl] (PPATBT-8) and poly(9,10-bis(4-(dodecyloxy)phenyl)-anthracene-2,6-diyl-alt-(5,6-bis(octyloxy)-4,7-di(2,2′-bithiophen-5-yl)benzo[c][1,2,5] thiadiazole)-5,5-diyl] (PPAT2BT-8), comprising 9,10-phenylanthracene as donor units and various 2,1,3-benzothiadiazole (BT) as acceptor units [21]. PPATBT, PPATBT-8, and PPAT2BT-8 have an E_g_ of 1.84, 1.96 and 1.86 eV, respectively. The photovoltaic performance of PPATBT is less than 2% because of its poor solubility. However, PPATBT-8 and PPAT2BT-8 have the PC efficiencies of 3.92 and 4.17% when mixed with PC_71_BM as the acceptor, respectively. Iraqi et al. also reported three new copolymers, poly[9,10-bis(triisopropylsilylacetylene)-anthracene-2,6-diyl-alt-(4,7-dithiophen-2-yl)-5,6-difluorobenzo[c][1,2,5]thiadiazole-5-5-diyl] (PTATffBT), poly[9,10-bis(triisopropylsilylacetylene)-anthracene-2,6-diylalt-(5,6-bis(octyloxy)-4,7-di(thiophene-2-yl)benzo[c]thiadiazole-5,5-diyl] (PTATBT-8) and poly(9,10-bis(triisopropylsilylacetylene)-anthracene-2,6-diylalt-(5,6-bis(octyloxy)-4,7-di(2,2′-bithiophen-5-yl)benzo[c][1,2,5]thiadiazole)-5,5-diyl] (PTAT2BT-8) [22]. The copolymers were synthesised by Suzuki polymerisation between 2,6-linked anthracene and various 2,1,3-benzothiadiazole acceptor units. Anthracene was functionalized with triisopropylsilylacetylene (TIPS) at its 9,10-positions. The E_g_ of the polymers are in the range of 1.81–1.92 eV. The PTATffBT has the deep-lying HOMO but high-lying LUMO energy levels compared to its PTATBT-8 and PTAT2BT-8 analogues. BHJ solar cell based on PTATBT-8: PC_71_BM has the PC efficiency of 2.36%. On the contrary, PTAT2BT-8 fabricated with PC_71_BM provides the higher PC efficiency of 3.15%.

Anthracene functionalized at 9,10-positions with dodecyloxy, dodecylthienyl and dodecylphenyl side chains were copolymerized through 2,6-positions with BT bearing dodecyloxy and octyloxy substituents yield POA12OTBT, PTA12OTBT, PBA12OTBT, POA8OTBT, PTA8OTBT and PBA8OTBT, respectively [19]. The E_g_ of the two dimensional (2D) conjugated polymers with thienyl and phenyl chains on anthracene unit are comparable (*ca*. 1.85 eV), which are much lower than those copolymers with dodecyloxy chains on the anthracene unit (*ca*. 2.13 eV). The 2D-conjugated polymers have deeper HOMO energy levels relative to those with dodecyloxy chains. Therefore, the *V*_oc_ values of the former polymers are higher than the latter polymers. The hole mobilities of POA12OTBT and POA8OTBT are lower than the polymers with the aromatic side group analogues. As a result, short-circuit current (*J*_sc_) values of the latter polymers are higher than the former polymers. POA8OTBT and POA12OTBT fabricated with PC_71_BM deliver the PC efficiencies of 1.82 and 2.26%, respectively. However, 2D-conjugated polymers provide the PC efficiency higher than 3% under the same experimental conditions. PBA8OTBT yields the highest PC efficiency of 4.34%.

Jo et al. reported four alternating copolymers, PTADTBT, PTADTFBT, PTADTDFBT and PTADTBTO. The copolymers were synthesised by Stille polymerisation between 9,10-thienylanthracene (TA) and BT with different substituents at 5,6-positions [13]. The E_g_ of the polymers are in the range of 1.8–2.1 eV and PTADTBTO has the highest E_g_ among all polymers prepared. The HOMO levels of the polymers are between −5.38 and −5.55 eV, while the PTADTDFBT exhibits the deepest HOMO energy level. The LUMO energy levels of PTADTBT, PTADTFBT and PTADTDFBT are comparable around −3.6 eV but PTADTBTO shows the highest LUMO level of −3.28 eV. PTADTBTO blended with PC_71_BM delivers the PC efficiency of 4.64%. However, PTADTBT and PTADTFBT blended with PC_71_BM provide the PC efficiency of 6.92 and 7.27%, respectively. PTADTDFBT yields the highest PC efficiency up to 8%.

Later on, Jo and co-workers further developed two novel D–A copolymers, PTATDPP and PTAFDPP, based on 9,10-thienyl substituted anthracene and DPP, respectively [23]. PTATDPP and PTAFDPP show the same E_g_ of 1.61 eV. They exhibit a high degree of crystallinity as confirmed by XRD studies. As a consequence, PTATDPP and PTAFDPP deliver high hole mobilities measured by FETs. PTAFDPP fabricated with PC_71_BM shows the PC efficiency of 5.22%. On the other hand, PTATDPP: PC_71_BM provides much higher PC efficiency up to 7% under the same experimental conditions.

**Scheme 1:**
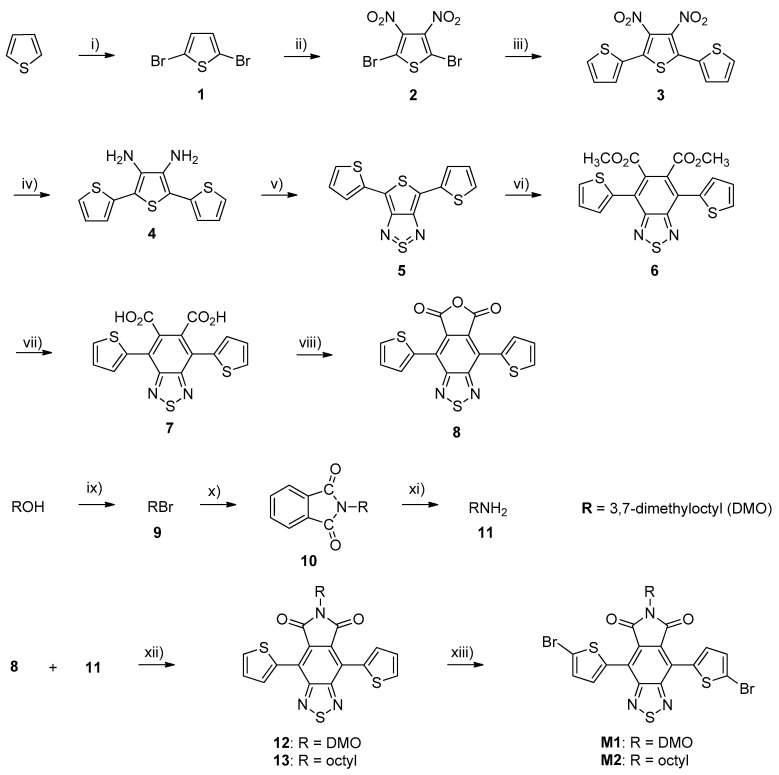
Synthetic steps of M1 and M2.

The synthetic methods for the preparation of benzothiadiazole dicarboxylic imide (BTDI) monomers (M1 and M2) are outlined in Scheme 1. M1 and M2 were synthesised through several steps starting from commercially available thiophene. For the synthesis of 2,5-dibromothiophene (1), thiophene was selectively brominated at 2,5-positions using two equivalents of *N*-bromosuccinimide (NBS) in *N*,*N*-dimethylformamide (DMF) in the dark to give material 1 as a yellow oily product in a high yield [24]. Then, material 1 was nitrated with concentrated nitric acid/sulfuric acid and fuming sulfuric acid to give 2,5-dibromo-3,4-dinitrothiophene (2) [25,26,27]. The compound 2 was then reacted with 2-(tributylstannyl)thiophene in the presence of PdCl_2_(PPh_3_)_2_ as a catalyst in anhydrous toluene at 115 °C to give 3′,4′-dinitro-2,2′:5′,2″-terthiophene (3). The resulting compound was obtained as orange crystals in a good yield of 90% [28]. 3′,4′-diamino-2,2′:5,2″-terthiophene (4) was obtained by a reduction reaction of material 3 using excess anhydrous tin (II) chloride (SnCl_2_). Material 4 was achieved as a brown solid in a good yield of 97% [29]. The resulting substance (4) was then reacted with *N*-thionyl aniline (PhNSO) and trimethylsilyl chloride (TMSCl) in anhydrous pyridine to afford 4,6-bis(2-thienyl)-thieno[3,4-*c*][1,2,5]-thiadiazole (5) as blue crystals in a good yield of 93% [30]. The compound 4,7-di(thien-2-yl)-2,1,3-benzothiadiazole-5,6-dimethyl ester (6) was obtained by the Diels–Alder reaction between material 5 and dimethyl acetylenedicarboxylate in anhydrous xylene at reflux. It was obtained in a good yield of 94% as yellow crystals [31]. Then, the material 6 was hydrolysed under basic conditions in ethanol under reflux followed by acidification to yield 4,7-di(thien-2-yl)-2,1,3-benzothiadiazole-5,6-dicarboxylic acid (7) as a yellow solid in a yield of 85% [32]. The compound 4,7-di(thien-2-yl)-2,1,3-benzothiadiazole-5,6-dicarboxylic anhydride (8) was synthesised by the intramolecular ring closure of material 7, in the presence of acetic anhydride and anhydrous xylene at 130 °C, to afford material 8 as a red solid in a good yield of 97% [33]. Moreover, 3,7-dimethyloctyl bromide (9) was synthesized from the reaction of commercially available 3,7-dimethyl-1-octanol with triphenylphosphine (Ph_3_P)/NBS in dichloromethane to yield material 9 as a colourless oil in 73% yield [34]. Then, material 9 was reacted with potassium phthalimide in anhydrous DMF to give *N*-(3,7-dimethyloctyl)phthalimide (10) as colourless oil in a good yield of 91% [35]. In addition, 3,7-dimethyl-1-octanamine (11) was obtained by Gabriel synthesis from the reaction of material 10 with hydrazine hydrate (NH_2_NH_2_) in methanol as a brown oil in 86% yield [36]. The compounds 4,7-di(thien-2-yl)-2,1,3-benzothiadiazole-5,6-*N*-(3,7-dimethyloctyl) dicarboxylic imide (12) and 4,7-di(thien-2-yl)-2,1,3-benzothiadiazole-5,6-*N*-octyl-dicarboxylic imide (13) were synthesized by the reaction of material 8 with material 11 and 1-octanamine in the presence of acetic acid and acetic anhydride to yield imide functionalized monomers (12 and 13) as orange solids in 84 and 93% yield, respectively. Lastly, the monomers 4,7-di(5-bromo-thien-2-yl)-2,1,3-benzothiadiazole-5,6-*N*-(3,7-dimethyloctyl)dicarboxylic imide (M1) and 4,7-di(5-bromo-thien-2-yl)-2,1,3-benzothiadiazole-5,6-*N*-octyl-dicarboxylic imide (M2) were prepared by the bromination of material 12 and material 13 at 5,5′-positions using NBS in tetrahydrofuran (THF) and to yield M1 and M2 as red solids in good yields of 98 and 96%, respectively.

Reagents and conditions:

i) NBS, DMF, −15 °C, room temperature, overnight;
ii) Fuming H_2_SO_4_ (20% free SO_3_), conc. H_2_SO_4_, conc. HNO_3_, 20 °C, 20–30 °C, 3 h;
iii) 2-(tributylstannyl)thiophene, anhydrous toluene, PdCl_2_(PPh_3_)_2_, 115 °C, 24 h;
iv) Anhydrous SnCl_2_, HCl (35%), ethanol, 30 °C, 24 h, NaOH (25%);
v) PhNSO, TMSCl, anhydrous pyridine, 3 h, RT, HCl (1.0 N);
vi) Dimethyl acetylenedicarboxylate, anhydrous xylene, reflux 24 h;
vii) Aqueous NaOH, ethanol, reflux 24 h, HCl (35%);
viii) Anhydrous Ac_2_O, anhydrous xylene, 130 °C, 6 h; ix) dichloromethane (DCM), PPh_3_, NBS, RT, 90 min;
ix) Potassium phthalimide, anhydrous DMF, 90 °C, 17 h, KOH;
x) Hydrazine hydrate (51%), methanol, reflux, HCl (5.0 M), reflux, 1 h;
xi) HOAc (100%), 100 °C overnight, Ac_2_O, 100 °C, 6 h;
xii) NBS, THF, RT, overnight.

**Scheme 2:**
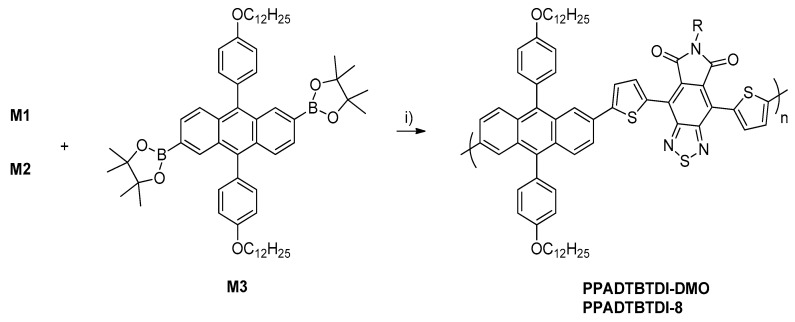
The synthesis of PPADTBTDI-DMO and PPADTBTDI-8 via Suzuki polymerisation.

**Figure 1:**
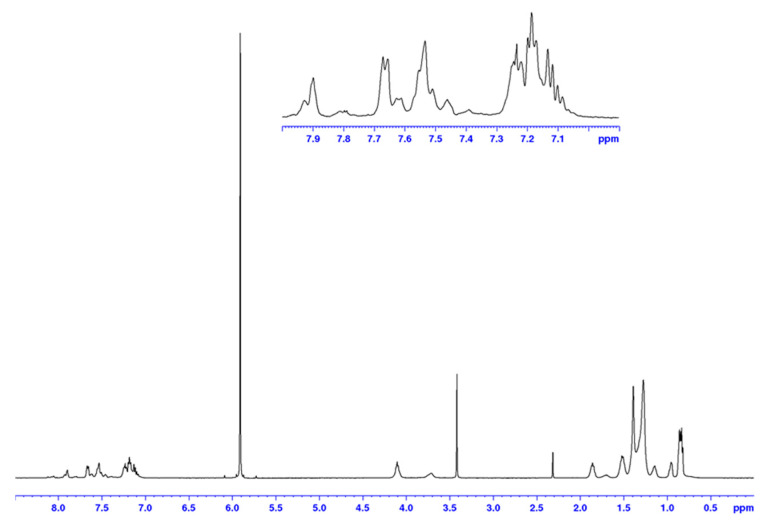
^1^ H NMR spectrum of PPADTBTDI-DMO in C_2_D_2_Cl_4_ at 100 °C.

**Figure 2:**
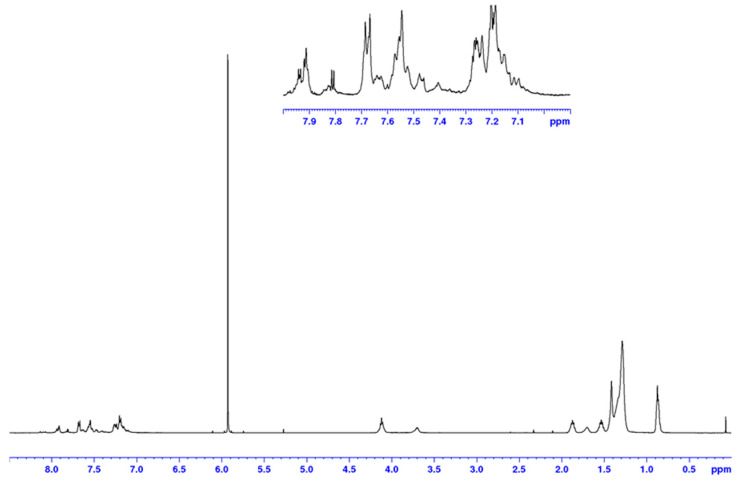
^1^ H NMR spectrum of PPADTBTDI-8 in C_2_D_2_Cl_4_ at 100 °C.

In this work, the preparation and characterisation of two alternating copolymers, PPADTBTDI-DMO and PPADTBTDI-8, are presented. The polymers were synthesised via the Suzuki polymerisation between the diboronic ester of 2,6-linked anthracene (M3) and dibrominated BTDI monomers (M1 and M2), respectively (Scheme 2). The polymerisations were performed with palladium(II) acetate [Pd(OAc)_2_]/tri(*o*-tolyl) phosphine [P(*o*-tol)_3_] catalyst, sodium hydrogen carbonate (NaHCO_3_) base in anhydrous THF [37]. Both polymerisations were left for 23–24 h with large amounts of purple precipitates forming as the reactions proceeded. The polymers were then dissolved in chloroform and an ammonia solution added then to the mixture stirred overnight to remove the palladium metal catalyst residues by forming tetraamminepalladium (II) dihydroxide [Pd(NH_3_)_4_(OH)_2_] soluble complexes. The polymers were obtained by precipitation from methanol followed by filtration. The polymers purified via Soxhlet extraction with methanol, acetone, hexane, toluene and finally chloroform. The small molecules, oligomers and impurities were removed in the methanol, acetone and hexane fractions. The toluene and chloroform fractions of the polymers were subsequently collected and concentrated in vacuo, re-precipitated in methanol followed by filtration to yield the purified polymers. The structures of PPADTBTDI-DMO and PPADTBTDI-8 were confirmed by the ^1^H NMR spectroscopy, FT-IR spectroscopy and elemental analysis. The ^1^H NMR spectra of the polymers are shown in Figure 1 and Figure 2.

Reagents and conditions: (i) anhydrous THF, NaHCO_3_, Pd(OAc)_2_, P(*o*-tol)_3_, 90 °C, 23–24 h.

## 2. Experimental Methodology

### 2.1. Materials

Thiophene (≥99%), *N*-bromosuccinimide (NBS) (99%), *N*,*N*-dimethylformamide (DMF) (≥99%), dichloromethane (DCM) (≥99.5%), 2-(tributylstannyl)thiophene (97%), bis(triphenylphosphine)palladium(II) dichloride [PdCl_2_(PPh_3_)_2_] (98%), *N*-thionylaniline (PhNSO) (98%), chlorotrimethylsilane [(CH_3_)_3_SiCl] (≥98%), pyridine (99.8%), xylene (≥99%), triphenylphosphine (Ph_3_P) (99%), 3,7-dimethyl-1-octanol (≥98%), potassium phthalimide(98%), hydrazine hydrate (50–60%), octylamine (99%), palladium(II) acetate [Pd(OAc)_2_] (98%), anhydrous acetic anhydride (≥99%), acetic acid (≥99%) and tris(*o*-tolyl)phosphine [P(o-tol)_3_] (97%) were purchased from Sigma-Aldrich (Gillingham, UK). Anhydrous tin(II) chloride (98%) and dimethyl acetylenedicarboxylate (98%) were purchased from Alfa Aesar (Heysham, UK). All of the starting materials and reagents obtained from Sigma-Aldrich and Alfa Aesar were utilized without further purification. The majority of the reactions were carried out under argon atmosphere. Anhydrous solvents used for the reactions obtained from Grubbs solvent purification system within the Sheffield University/Chemistry department. 9,10-Bis(4-(dodecyloxy)phenyl)-2,6-bis(4,4,5,5-tetramethyl-1,3,2-dioxaborolan-2-yl) anthracene (M3) [21] was prepared according to literature procedure.

### 2.2. Measurements

^1^ H and ^13^ C nuclear magnetic resonance (NMR) spectra for the monomers measured either with a Bruker Avance AV 3HD 400 (400 MHz) spectrometer (Bruker, Berlin, Germany) in deuterated chloroform (CDCl_3_), deuterated acetone (CD_3_COCD_3_) or deuterated dimethyl sulfoxide (CD_3_SOCD_3_) as solvents at room temperature. The ^1^H NMR spectra for the polymers were measured with Bruker AV 3HD 500 (Bruker, Coventry, United Kingdom) (500 MHz) in deuterated 1,1,2,2-tetrachloroethane (C_2_D_2_Cl_4_) as the solvent at 100 °C. The chemical shifts were measured in parts per million (ppm) relative to tetramethylsilane (δ_H_ 0.00). The coupling constants (*J*) are calculated in Hertz (Hz). The ^1^H and ^13^C NMR spectra were analysed using Bruker TopSpin 3.2 software. Carbon, hydrogen, nitrogen and sulphur elemental analysis was performed by either the Perkin Elmer 2400 CHNS/O Series II Elemental Analyser (Perkin Elmer, Buckinghamshire, United Kingdom) or Vario MICRO Cube CHN/S Elemental Analyser for carbon, hydrogen, and nitrogen (CHN) analysis. Halides analysis (Br, F, and I) was performed by the Schöniger oxygen flask combustion method. Mass spectra for the monomers were recorded on Agilent 7200 accurate mass Q-TOF GC–MS spectrometer (Agilent, Santa Clara, CA, USA). Helium is used as a carrier gas in rate of (1.2 mL min^−1^), the injection volume is (1.0 μL) and the concentration of the measured sample is (5 mg mL^−1^) in the CHCl_3_ solvent. The temperature program is between 60 and 320 °C at 10 °C min^−1^. Mass spectra for the monomers were obtained by the electron ionization method (EI). Gel permeation chromatography (GPC) measurements accomplished by Viscotek GPC Max, a waters 410 instrument with a differential refractive index detector, two Polymer Labs PLgel 5μ Mixed C (7.5 × 300 mm) columns and a guard (7.5 × 50 mm). Molecular weights for the polymers were determined by preparing polymer solutions (2.5 mg mL^−1^) using chloroform (HPLC grade). The columns were thermostated at 40 °C using CHCl_3_. GPC curves were calibrated using a series of narrow polystyrene standards (Polymer Laboratories). Ultraviolet–visible (UV–vis) absorption spectra were measured by SPECORD S600 Double Beam UV/visible Spectrophotometer (Hitachi, Berkshire, United Kingdom) at room temperature. The absorbance of the polymers was measured in CHCl_3_ solution using quartz cuvettes (light path length = 10 mm) and blank quartz cuvettes including CHCl_3_ was used as a reference. The polymers were coated on quartz substrates from CHCl_3_ solutions (1 mg mL^−1^) and blank quartz substrate was used as a reference. Thermogravimetric analysis (TGA) measurements were recorded by Perkin Elmer (Pyris 1) Thermogravimetric Analyser (Perkin Elmer, Buckinghamshire, United Kingdom) at a scan rate of 10 °C min^−1^ under an inert nitrogen atmosphere. Platinum pans were used as the sample holder and the weight of the measured samples was about (3 mg). XRD for the polymers was measured by Bruker D8 ADVANCE X-ray powder diffractometer with a CuKα radiation source (0.15418 nm, rated as 1.6 kW). The scanning angle was conducted over the range 2°–40°. Cyclic voltammograms were recorded using a Princeton Applied Research Model 263A Potentiostat/Galvanostat (Princeton Applied Research, Cambridge, United Kingdom). A three-electrode system was used containing a Pt disc (area = 3.14 × 10^−2^ cm^2^), platinum wire and Ag/Ag^+^ as the working electrode, counter electrode and reference electrode, respectively, in tetrabutylammonium perchlorate (Bu_4_NClO_4_) acetonitrile solution (0.1 mol dm^−3^). Measurements were conducted on polymer films that were prepared by drop casting 1.0 mm^3^ of polymer solution (1 mg cm^−2^ in chloroform (HPLC grade)). Infrared absorption spectra were recorded on ATR Perkin Elmer Rx/FT-IR system and Nicolet Model 205 FT-IR spectrometer.

### 2.3. Monomers and Polymers Synthesis

#### 2.3.1. Synthesis of 2,5-dibromothiophene (1)

The following is a modified procedure of previously reported by Ponomarenko et al. [24]. Thiophene (25.00 g, 297.12 mmol) in *N*,*N*-dimethylformamide (DMF) (250 mL) was added to a flask and cooled to −15 °C. To this solution, *N*-bromosuccinimide (NBS) (110.00 g, 618.04 mmol) in DMF (300 mL) was added dropwise in the dark and the reaction was stirred overnight at RT. The reaction contents were put into ice and dichloromethane (DCM) and subsequently extracted with DCM and the organic phase was washed with deionized H_2_O to a neutral pH. The organic layer was collected and dried over MgSO_4_ and the solvent was concentrated to afford the product which was purified by vacuum distillation and obtained material 1 as a yellow oil (59.30 g, 245 mmol, 82% yield). ^1^H NMR (CDCl_3_, δ): 6.87 (s, 2H). ^13^C NMR (CDCl_3_, δ): 130.4, 111.6. FT-IR (cm^−1^): 3096, 1726, 1516, 1410, 1200. EI–MS (m/z): 242 [M]^+^. Elemental analysis (%) calculated for C_4_H_2_Br_2_S: C, 19.86; H, 0.83; Br, 66.06, S, 13.25. Found: C, 20.01; H, 0.85; Br, 65.02, S, 11.96.

#### 2.3.2. Synthesis of 2,5-dibromo-3,4-dinitrothiophene (2)

Material 2 was prepared according to the modified procedure as reported by Wen and Rasmussen [25]. Concentrated H_2_SO_4_ (150 mL) and fuming H_2_SO_4_ (150 mL, 20% free SO_3_) were combined in a flask. This flask was cooled to 0 °C and material 1 (26.00 g, 107.46 mmol) was added dropwise. Concentrated nitric acid (125 mL) was added dropwise and the reaction contents were kept under 20 °C. During the addition of nitric acid, yellow precipitate formed quickly. The mixture was stirred for 3 h at 20–30 °C. Then, the mixture was poured into an ice and upon the melting of the ice, a yellow precipitate was filtrated and washed thoroughly with deionized H_2_O. The product recrystallized from methanol to afford material 2 as yellow crystals (32.50 g, 98 mmol, 91% yield). ^13^C NMR (CDCl_3_, δ): 140.7, 113.4. FT-IR (cm^−1^): 2886, 2851, 2813, 1535, 1497, 1345, 1081. EI–MS (*m*/*z*): 332 [M]^+^. Elemental analysis (%) calculated for C_4_Br_2_N_2_O_4_S: C, 14.47; N, 8.44; S, 9.66; Br, 48.15. Found: C, 14.51; N, 7.91; S, 9.19; Br, 46.57.

#### 2.3.3. Synthesis of 3′,4′-dinitro-2,2′:5′,2″-terthiophene (3)

Material 3 was prepared according to the modified established procedure as documented by Schwiderski and Rasmussen [28]. In a flask, material 2 (9.90 g, 29.82 mmol), 2-(tributylstannyl)thiophene (27.82 g, 74.54 mmol) and bis(triphenylphosphine)palladium(II) dichloride [PdCl_2_(PPh_3_)_2_] (0.45 g, 0.64 mmol) were added. The system degassed under argon and anhydrous toluene (100 mL) was added and heated at 115 °C for 24 h. The flask cooled to RT and the volatiles were removed to obtain the product which was purified by column chromatography with the gradient (petroleum ether, 0–50% DCM) to obtain an orange solid and the product was further purified by recrystallization from methanol to obtain material 3 as orange crystals (9.10 g, 27 mmol, 90% yield). ^1^H NMR (CDCl_3_, δ): 7.62 (dd, 2H, J = 1.0 Hz, 5.0 Hz), 7.56 (dd, 2H, J = 1.0 Hz, 4.0 Hz), 7.19 (dd, 2H, J = 4.0 Hz, 5.0 Hz). ^13^C NMR (CDCl_3_, δ): 135.9, 133.9, 131.3, 131.2, 128.4, 128.0. FT-IR (cm^−1^): 3076, 1821, 1528, 1379, 1348, 1299, 1223, 1066. EI–MS (*m*/*z*): 338 [M]^+^. Elemental analysis (%) calculated for C_12_H_6_N_2_O_4_S_3_: C, 42.60; H, 1.79; N, 8.28; S, 28.42. Found: C, 42.49; H, 1.66; N, 8.13; S, 28.16.

#### 2.3.4. Synthesis of 3′,4′-diamino-2,2′:5,2″-terthiophene (4)

Material 4 was prepared according to the modified procedure as reported by Hailu et al. [29]. EtOH (31 mL) and HCl (62 mL, 35%) were added to material 3 (3.00 g, 8.86 mmol) in a flask. To this mixture, anhydrous tin(II) chloride (31.00 g, 163.50 mmol) in ethanol (62 mL) was added and stirred at 30 °C for 24 h. The mixture was cooled to RT and put into cold NaOH. To this mixture, toluene was added and then stirred vigorously and filtered through celite. The product extracted with toluene and the organic phases was washed with NaCl and subsequently dried over MgSO_4_. The solvent was concentrated to obtain material 4 as a brown solid (2.40 g, 9 mmol, 97% yield). ^1^H NMR (CDCl_3_, δ): 7.30 (d, 2H, J = 2.0 Hz), 7.27 (s, 2H), 7.09–7.14 (m, 2H), 3.76 (bs, 4H). ^13^C NMR (CDCl_3_, δ): 136.0, 133.6, 127.8, 124.0, 124.0, 110.1. FT-IR (cm^−1^): 3371, 3298, 3224, 3182, 3096, 1631, 1615, 1573, 1528, 1509, 1441, 1336, 1294, 1070. EI–MS (*m*/*z*): 278 [M]^+^. Elemental analysis (%) calculated for C_12_H_10_N_2_S_3_: C, 51.77; H, 3.62; N, 10.06; S, 34.55. Found: C, 51.69; H, 3.54; N, 9.97; S, 34.78.

#### 2.3.5. Synthesis of 4,6-bis(2-thienyl)-thieno[3,4-c][1,2,5]-thiadiazole (5)

Material 5 was prepared according to the modified established procedure as reported by Delgado et al. [30]. Material 4 (1.67 g, 5.99 mmol) was dissolved in dry pyridine (30 mL) in a flask and degassed under argon. To this mixture, *N*-thionylaniline (1.60 g, 11.49 mmol) was added dropwise and chlorotrimethylsilane (4.50 g, 41.42 mmol) was then added dropwise, resulting in a dark blue colour. The reaction contents were stirred for 3 h at RT and then put into DCM. The solution was washed with HCl and with deionized water and extracted with DCM. The organic phase dried over anhydrous MgSO_4_ and was subsequently filtered. The solvent evaporated to otain the product which was purified via chromatography with DCM to produce material 5 as blue crystals (1.72 g, 6 mmol, 93% yield). ^1^H NMR (CDCl_3_, δ): 7.59 (dd, 2H, J = 1.0 Hz, 3.5 Hz), 7.34 (dd, 2H, J = 1.0 Hz, 5.0 Hz), 7.12 (dd, 2H, J = 3.5 Hz, 5.0 Hz). 13C NMR (CDCl_3_, δ): 156.3, 135.0, 128.2, 125.4, 124.3, 112.4. FT-IR (cm^−1^): 3102, 3073, 1797, 1525, 1483, 1365, 1223, 1137, 1047. EI–MS (*m*/*z*): 306 [M]^+^. Elemental analysis (%) calculated for C_12_H_6_N_2_S_4_: C, 47.04; H, 1.97; N, 9.14; S, 41.85. Found: C, 47.25; H, 2.18; N, 8.83; S, 39.16.

#### 2.3.6. Synthesis of 4,7-di(thien-2-yl)-2,1,3-benzothiadiazole-5,6-dimethyl ester (6)

Material 6 was prepared according to the modified procedure as documented by Wang et al. [31]. Material 5 (1.86 g, 6.06 mmol) and dimethyl acetylenedicarboxylate (1.73 g, 12.17 mmol) were combined in a flask. The system was evacuated and refilled with argon for three cycles before anhydrous xylene (40 mL) was added. The reaction contents were refluxed for 24 h. The flask cooled to RT and the solvent was removed to afford the product which was purified by column chromatography with gradient (petroleum ether, 0–50% DCM) to obtain material 6 as yellow crystals (2.37 g, 6 mmol, 94% yield). ^1^H NMR (CDCl_3_, δ): 7.62 (dd, 2H, J = 1.0 Hz, 5.0 Hz), 7.44 (dd, 2H, J = 1.0 Hz, 3.5 Hz), 7.22 (dd, 2H, J = 3.5 Hz, 5.0 Hz), 3.78 (s, 6H). ^13^C NMR (CDCl_3_, δ): 168.1, 153.6, 135.1, 132.0, 129.7, 129.0, 127.3, 126.2, 53.1. FT-IR (cm^−1^): 3109, 2975, 2932, 2900, 2865, 2159, 2031, 1971, 1730, 1513, 1460, 1318, 1283, 1198. EI–MS (*m*/*z*): 416 [M]^+^. Elemental analysis (%) calculated for C_18_H_12_N_2_O_4_S_3_: C, 51.91; H, 2.90; N, 6.73; S, 23.09. Found: C, 51.86; H, 2.94; N, 6.61; S, 22.97.

#### 2.3.7. Synthesis of 4,7-di(thien-2-yl)-2,1,3-benzothiadiazole-5,6-dicarboxylic acid (7)

Material 7 was prepared according to the modified established procedure as reported by Nielsen et al. [32]. Sodium hydroxide (4.00 g, 100.00 mmol) dissolved in deionized water (30 mL) and added to a flask. To this solution, ethanol (200 mL) and material 6 (2.27 g, 5.45 mmol) were added and the reaction contents were refluxed for 24 h. The flask cooled to RT and deionized H_2_O was added. This mixture cooled to 0 °C and was neutralized by HCl to precipitate the product. The precipitate was filtered and subsequently washed with deionized H_2_O. The precipitate dried under high vacuum to obtain material 7 as a yellow solid (1.80 g, 5 mmol, 85% yield). ^1^H NMR (CD_3_SOCD_3_, δ): 7.86 (dd, 2H, J = 1.0 Hz, 5.0 Hz), 7.47 (dd, 2H, J = 1.0 Hz, 3.5 Hz), 7.25 (dd, 2H, J = 3.5 Hz, 5.0 Hz). ^13^C NMR (CD_3_SOCD_3_, δ): 168.4, 152.5, 134.8, 133.0, 129.7, 129.3, 127.2, 123.8. FT-IR (cm^−1^): 3106, broad (3300–2600), 2162, 2024, 1971, 1815, 1765, 1705, 1552, 1453, 1386, 1261, 1152, 1020. EI–MS (*m*/*z*): 387 [M − H]^+^. Elemental analysis (%) calculated for C_16_H_8_N_2_O_4_S_3_: C, 49.48; H, 2.08; N, 7.21; S, 24.76. Found: C, 45.33; H, 2.70; N, 6.47; S, 21.35.

#### 2.3.8. Synthesis of 4,7-di(thien-2-yl)-2,1,3-benzothiadiazole-5,6-dicarboxylic anhydride (8)

Material 8 was prepared according to the modified procedure as documented by Lan et al. [33]. Material 7 (1.15 g, 2.96 mmol) and anhydrous acetic anhydride (10.00 g, 97.95 mmol) were combined in a flask. The system was evacuated and refilled with argon for three cycles before anhydrous xylene (30 mL) was added. The mixture was heated at 130 °C for 6 h. The mixture cooled to RT, and the solvent evaporated to obtain material 8 as red solid (1.06 g, 3 mmol, 97% yield). ^1^H NMR (CDCl_3_, δ): 8.11 (dd, 2H, J = 1.0 Hz, 4.0 Hz), 7.82 (dd, 2H, J = 1.0 Hz, 5.0 Hz), 7.33 (dd, 2H, J = 4.0 Hz, 5.0 Hz). ^13^C NMR (CD_3_SOCD_3_, δ): 162.0, 156.0, 134.3, 132.6, 131.4, 127.8, 127.6, 125.5. FT-IR (cm^−1^): 3131, 3109, 3081, 1808, 1765, 1552, 1453, 1393, 1247, 1152, 1088. EI–MS (m/z): 370 [M]^+^. Elemental analysis (%) calculated for C_16_H_6_N_2_O_3_S_3_: C, 51.88; H, 1.63; N, 7.56; S, 25.97. Found: C, 52.11; H, 2.00; N, 7.20; S, 24.55.

#### 2.3.9. Synthesis of 3,7-dimethyloctyl bromide (9)

Material 9 was prepared according to the modified procedure as indicated by Matsueda et al. [34]. Triphenylphosphine (21.10 g, 80.44 mmol) was added to a mixture of 3,7-dimethyl-1-octanol (12.61 g, 79.69 mmol) and dichloromethane (250 mL) and stirred in a flask. To this mixture, NBS (14.26 g, 80.14 mmol) was added portionwise and stirred at RT for 90 min. The mixture was washed with NaHCO_3_ solution, dried over MgSO_4_, filtered and the solvent was evaporated. The substance was stirred in petroleum ether for 1 h at RT, filtered and the filtrate evaporated. The product was purified by chromatography with petroleum ether to yield material 9 as colourless oil (23.00 g, 59 mmol, 73% yield). ^1^ H NMR (CDCl_3_, δ): 3.55–3.37 (m, 2H), 1.96–1.83 (m, 1H), 1.77–1.61 (m, 2H), 1.60–1.49 (m, 1H), 1.41–1.24 (m, 3H), 1.22–1.11 (m, 3H), 0.82–0.94 (m, 9H). ^13^C NMR (CDCl_3_, δ): 40.1, 39.2, 36.7, 32.3, 31.7, 28.0, 24.6, 22.7, 22.6, 19.0. FT-IR (cm^−1^): 2953, 2925, 2868, 1464, 1382, 1261, 1173. EI–MS (*m*/*z*): 222.1 [M]^+^. Elemental analysis (%) calculated for C_10_H_21_Br: C, 54.30; H, 9.57; Br, 36.13. Found: C, 55.04; H, 9.53; Br, 34.23.

#### 2.3.10. Synthesis of N-(3,7-dimethyloctyl) phthalimide (10)

Material 10 was prepared according to the modified procedure as reported by Thomson et al. [35]. Material 9 (4.07 g, 18.40 mmol) and anhydrous DMF (20 mL) were added in a flask. To this mixture, potassium phthalimide (3.75 g, 20.27 mmol) was added and the reaction contents were heated to 90 °C for 17 h. The mixture was cooled to RT and put in deionized H_2_O and the product was subsequently extracted with DCM. The organic extracts combined, washed with KOH and deionized water. The organic phase dried over MgSO_4_ and the solvent was evaporated to obtain the product which was purified via chromatography with dichloromethane to yield material 10 as a colourless oil (5.29 g, 18 mmol, 91% yield). ^1^H NMR (CDCl_3_, δ): 7.85 (dd, 2H, J = 3.0 Hz, 5.5 Hz), 7.72 (dd, 2H, J = 3.0 Hz, 5.5 Hz), 3.80–3.66 (m, 2H), 1.77–1.66 (m, 1H), 1.53–1.43 (m, 3H), 1.41–1.25 (m, 3H), 1.20–1.11 (m, 3H), 0.98 (d, 3H, J = 6.5 Hz), 0.87 (d, 6H, J = 7.0 Hz). ^13^C NMR (CDCl_3_, δ): 168.4, 133.8, 132.2, 123.1, 39.2, 37.0, 36.3, 35.5, 30.7, 27.9, 24.5, 22.7, 22.6, 19.4. FT-IR (cm^−1^): 2953, 2925, 2868, 1772, 1706, 1616, 1469, 1398, 1267, 1189, 1055. EI–MS (*m*/*z*): 288.2 [MH]^+^. Elemental analysis (%) calculated for C_18_H_25_NO_2_: C, 75.22; H, 8.77; N, 4.87. Found: C, 72.17; H, 8.62; N, 4.43.

#### 2.3.11. Synthesis of 3,7-dimethyl-1-octanamine (11)

Material 11 was prepared according to the modified procedure as documented by Yue et al. [36]. Material 10 (6.03 g, 20.98 mmol), hydrazine hydrate (4.0 mL, 65.0 mmol, 51%) and methanol (100 mL) were combined in a flask. The reaction contents refluxed until the starting material disappeared. Upon completion, excess HCl was added and the mixture was refluxed for 1 h and then cooled to RT. The precipitate was filtered and washed with water. The methanol was concentrated and the residue was diluted with dichloromethane. The organic layer was washed with KOH and the product was extracted with dichloromethane. The organic phase was washed with NaCl, dried over MgSO_4_ and the solvent was concentrated to yield material 11 as a brown oil (2.85 g, 18 mmol, 86% yield). ^1^H NMR (CDCl_3_, δ): 2.82–2.62 (m, 2H), 1.60–1.43 (m, 3H), 1.35–1.22 (m, 4H), 1.20–1.06 (m, 3H), 0.88 (dd, 9H, J = 2.0 Hz, 6.5 Hz). ^13^C NMR (CDCl_3_, δ): 41.1, 40.1, 39.3, 37.3, 30.5, 28.0, 24.7, 22.7, 22.6, 19.6. FT-IR (cm^−1^): 3521, 3375, 3219, 3021, 2953, 2925, 2868, 2155, 2028, 1978, 1598, 1464, 1382, 1166, 1063. EI–MS (*m*/*z*): 157.2 [M]^+^. Elemental analysis (%) calculated for C_10_H_23_N: C, 76.36; H, 14.74; N, 8.90. Found: C, 71.74; H, 13.51; N, 7.71.

#### 2.3.12. Synthesis of 4,7-di(thien-2-yl)-2,1,3-benzothiadiazole-5,6-*N*-(3,7-dimethyloctyl)dicarboxylic imide (12)

Material 8 (1.00 g, 2.69 mmol), acetic acid (50 mL, 100%) and material 11 (0.88 g, 5.59 mmol) were combined in a flask. The system was evacuated and refilled with argon for three cycles and heated at 110 °C overnight. The mixture was cooled to RT, acetic anhydride (20 mL) was added and heated at 110 °C for 6 h. The mixture was cooled to RT and the solvent was concentrated to yield the product which was purified by chromatography with (60:10, petroleum ether: ethyl acetate) to afford material 12 as an orange solid (1.15 g, 2.3 mmol, 84% yield). ^1^H NMR (CDCl_3_, δ): 7.91 (dd, 2H, J = 1.0 Hz, 3.5 Hz), 7.73 (dd, 2H, J = 1.0 Hz, 5.0 Hz), 7.30 (dd, 2H, J = 3.5 Hz, 5.0 Hz), 3.84–3.70 (m, 2H), 1.78–1.65 (m, 1H), 1.55–1.43 (m, 3H), 1.39–1.22 (m, 3H), 1.20–1.08 (m, 3H), 0.97 (d, 3H, J = 6.0 Hz), 0.86 (d, 6H, J = 6.0 Hz). ^13^C NMR (CDCl_3_, δ): 165.7, 156.5, 133.1, 131.5, 130.2, 127.0, 126.9, 126.7, 39.2, 37.2, 37.0, 35.2, 31.0, 27.9, 24.6, 22.7, 22.6, 19.4. FT-IR (cm^−1^): 3439, 3102, 3074, 2953, 2925, 2865, 1804, 1751, 1694, 1549, 1453, 1364, 1226, 1162, 1056. EI–MS (*m*/*z*): 510.1 [MH]^+^. Elemental analysis (%) calculated for C_26_H_27_N_3_O_2_S_3_: C, 61.27; H, 5.34; N, 8.24; S, 18.87. Found: C, 61.59; H, 5.56; N, 7.94; S, 16.79.

#### 2.3.13. Synthesis of 4,7-di(thien-2-yl)-2,1,3-benzothiadiazole-5,6-*N*-octyl-dicarboxylic imide (13)

Material 13 was prepared followed by the same procedure for the synthesis of material 12 except octylamine (1.20 g, 9.28 mmol) was used. Material 13 was obtained as an orange solid (1.20 g, 2.5 mmol, 93% yield). ^1^H NMR (CDCl_3_, δ): 7.91 (dd, 2H, J = 1.0 Hz, 3.5 Hz), 7.73 (dd, 2H, J = 1.0 Hz, 5.0 Hz), 7.30 (dd, 2H, J = 3.5 Hz, 5.0 Hz), 3.74 (t, 2H, J = 7.5 Hz), 1.65–1.76 (m, 2H), 1.23–1.41 (m, 10H), 0.88 (t, 3H, J = 7.0 Hz). ^13^C NMR (CDCl_3_, δ): 165.8, 156.5, 133.1, 131.5, 130.2, 127.1, 126.9, 126.7, 39.0, 31.8, 29.1, 28.2, 27.0, 22.7, 14.0. FT-IR (cm^−1^): 3443, 3102, 3070, 2918, 2854, 1808, 1754, 1694, 1556, 1457, 1364, 1226, 1169, 1098. EI–MS (*m*/*z*): 481.1 [M]^+^. Elemental analysis (%) calculated for C_24_H_23_N_3_O_2_S_3_: C, 59.85; H, 4.81; N, 8.72; S, 19.97. Found: C, 59.91; H, 4.93; N, 8.70; S, 20.72.

#### 2.3.14. Synthesis of 4,7-di(5-bromo-thien-2-yl)-2,1,3-benzothiadiazole-5,6-*N*-(3,7-dimethyloctyl)dicarboxylic imide (M1)

Material 12 (1.00 g, 1.96 mmol) and THF (100 mL) were combined in a flask. To this mixture, NBS (1.74 g, 9.77 mmol) was added and stirred at RT overnight in the dark. The solvent evaporated to obtain the product as a red solid, and subsequently was washed with cold CH_3_OH, filtered and dried. The product was purified via chromatography with DCM to yield M1 as a red solid (1.28 g, 2 mmol, 98% yield). ^1^H NMR (CDCl_3_, δ): 7.80 (d, 2H, J = 4.0 Hz), 7.24 (d, 2H, J = 4.0 Hz), 3.70–3.84 (m, 2H), 1.78–1.66 (m, 1H), 1.54–1.44 (m, 3H), 1.41–1.22 (m, 3H), 1.20–1.11 (m, 3H), 0.98 (d, 3H, J = 6.0 Hz), 0.87 (d, 6H, J = 6.5 Hz). ^13^C NMR (CDCl_3_, δ): 165.6, 155.9, 134.1, 133.0, 129.8, 126.4, 125.8, 118.7, 39.2, 37.3, 37.0, 35.2, 31.0, 27.9, 24.6, 22.7, 22.6, 19.4. FT-IR (cm^−1^): 3429, 3120, 2957, 2918, 2865, 1747, 1691, 1563, 1460, 1364, 1283, 1073. EI–MS (*m*/*z*): 666.9 [M]^+^. Elemental analysis (%) calculated for C_26_H_25_Br_2_N_3_O_2_S_3_: C, 46.78; H, 3.78; Br, 23.94; N, 6.30; S, 14.41. Found: C, 46.61; H, 3.61; Br, 23.95; N, 6.29; S, 14.64.

#### 2.3.15. Synthesis of 4,7-di(5-bromo-thien-2-yl)-2,1,3-benzothiadiazole-5,6-*N*-octyl-dicarboxylic imide (M2)

M2 was prepared followed by the same procedure for the synthesis of M1, where compound material 13 (1.00 g, 2.07 mmol) was used with THF (100 mL) and NBS (1.84 g, 10.33 mmol). M2 was obtained as a red solid (1.27 g, 2 mmol, 96% yield). ^1^H NMR (CDCl_3_, δ): 7.80 (d, 2H, J = 4.0 Hz), 7.24 (d, 2H, J = 4.0 Hz), 3.75 (t, 2H, J = 7.0 Hz,), 1.66–1.75 (m, 2H), 1.23–1.40 (m, 10H), 0.88 (t, 3H, J = 6.5 Hz,). ^13^C NMR (CDCl_3_, δ): 165.7, 156.0, 134.1, 133.0, 129.8, 126.4, 125.9, 118.7, 39.0, 31.8, 29.1, 28.3, 27.0, 22.6, 14.1. FT-IR (cm^−1^): 3421, 3120, 2953, 2911, 2850, 1744, 1687, 1556, 1446, 1375, 1244, 1176. EI–MS (*m*/*z*): 638.9 [M]^+^. Elemental analysis (%) calculated for C_24_H_21_Br_2_N_3_O_2_S_3_: C, 45.08; H, 3.31; N, 6.57; S, 15.04; Br, 24.99. Found: C, 44.79; H, 3.41; N, 6.47; S, 15.74; Br, 28.80.

#### 2.3.16. Poly[9,10-bis(4-(dodecyloxy)phenyl)-2,6-anthracene-alt-5,5-(4′,7′-bis(2-thienyl)-2′,1′,3′-benzothiadiazole-*N*-5,6-(3,7-dimethyloctyl)dicarboxylic imide)] (PPADTBTDI-DMO)

M1 (150 mg, 0.224 mmol) and M3 (213.6 mg, 0.224 mmol) were added to a flask and degassed under argon. Anhydrous THF (10 mL) followed by sodium hydrogen carbonate solution (2.5 mL, 5% wt, degassed) were added and the system was degassed again. To this mixture, Pd(OAc)_2_ (3.7 mg, 0.0168 mmol) and P(*o*-tol)_3_ (10.2 mg, 0.0336 mmol) were added, degassed and heated at 90 °C for 23 h. The flask cooled to RT, the polymer was dissolved in CHCl_3_ (200 mL) and an NH_4_OH solution (50 mL, 35% in H_2_O) was added and the mixture was stirred overnight. The organic phase was separated and washed with deionized H_2_O. The organic phase was concentrated to around (50 mL) and put into methanol (300 mL) and stirred overnight. The mixture was filtered and the polymer was cleaned using Soxhlet extraction with methanol (300 mL), acetone (300 mL), hexane (300 mL) and then toluene (300 mL). The toluene fraction was concentrated to around (50 mL) and then put into methanol (300 mL). The mixture was stirred overnight and the pure polymer recovered by filtration to obtain PPADTBTDI-DMO as purple powders. Toluene fraction (82 mg, 0.07 mmol, 30% yield), chloroform fraction (180 mg, 0.15 mmol, 67% yield) with total yield 97%. GPC: toluene fraction, *M*_n_ = 6700 g mol^−1^, *M*_w_ = 12600 g mol^−1^, PDI = 1.8 and Dp = 6; chloroform fraction, *M*_n_ = 12700 g mol^−1^, *M*_w_ = 22400 g mol^−1^, PDI = 1.7 and Dp = 11. ^1^H NMR (toluene fraction) (C_2_D_2_Cl_4_, δ): 7.96–7.87 (bm, 2H), 7.71–7.61 (bm, 2H), 7.57–7.50 (bm, 4H), 7.30–7.05 (bm, 10H), 4.18–4.01 (bm, 4H), 3.81–3.61 (bm, 2H), 1.91–1.79 (bm, 4H), 1.78–1.64 (bm, 1H), 1.60–1.46 (bm, 8H), 1.43–1.21 (bm, 34H), 1.20–1.09 (bm, 3H), 1.00–0.90 (bm, 3H), 0.89–0.78 (bm, 12H). FT-IR (cm^−1^): 2918, 2850, 1754, 1701, 1606, 1570, 1428, 1361, 1240, 1176, 1066. Elemental analysis (%) calculated for C_76_H_89_N_3_O_4_S_3_: C, 75.77; H, 7.45; N, 3.49; S, 7.98. Found: C, 73.30; H, 7.15; N, 3.27; S, 7.79.

#### 2.3.17. Poly[9,10-bis(4-(dodecyloxy)phenyl)-2,6-anthracene-alt-5,5-(4′,7′-bis(2-thienyl)-2′,1′,3′-benzothiadiazole-5,6-*N*-octyl-dicarboxylic imide)] (PPADTBTDI-8)

M2 (143.2 mg, 0.224 mmol) and M3 (213.6 mg, 0.224 mmol) were added to a flask and degassed under argon. Anhydrous THF (10 mL) followed by sodium hydrogen carbonate solution (2.5 mL, 5% wt, degassed) were added and the system, which was degassed again. To this mixture, Pd(OAc)_2_ (3.7 mg, 0.0168 mmol) and P(*o*-tol)_3_ (10.2 mg, 0.0336 mmol) were added, degassed and heated at 90 °C for 24 h. The flask was cooled to RT, the polymer was dissolved in CHCl_3_ (200 mL) and an NH_4_OH solution (50 mL, 35% in H_2_O) was added and the mixture stirred overnight. The organic phase was separated and washed with deionized H_2_O. The organic phase concentrated to around (50 mL) and put into methanol (300 mL) and stirred overnight. The mixture filtered and the polymer cleaned using Soxhlet extraction with methanol (300 mL), acetone (300 mL), hexane (300 mL) and then toluene (300 mL). The toluene fraction was concentrated to around (50 mL) and was then put into methanol (300 mL). The mixture was stirred overnight and the pure polymer was recovered by filtration to afford PPADTBTDI-8 as purple powders. Toluene fraction (65 mg, 0.05 mmol, 25% yield), chloroform fraction (190 mg, 0.16 mmol, 72% yield) with total yield 97%. GPC: toluene fraction, *M*_n_ = 6000 g mol^−1^, *M*_w_ = 11100 g mol^−1^, PDI = 1.8 and Dp = 5; chloroform fraction, *M*_n_ = 12500 g mol^−1^, *M*_w_ = 27400 g mol^−1^, PDI = 2.1 and Dp = 11. ^1^H NMR (toluene fraction) (C_2_D_2_Cl_4_, δ): 7.96–7.87 (bm, 2H), 7.71–7.61 (bm, 2H), 7.57–7.50 (bm, 4H), 7.30–7.05 (bm, 10H), 4.18–4.01 (bm, 4H), 3.81–3.61 (bm, 2H), 1.91–1.79 (bm, 4H), 1.79–1.63 (bm, 2H), 1.60–1.48 (bm, 4H), 1.46–1.20 (bm, 42H), 0.92–0.79 (bm, 9H). FT-IR (cm^−1^): 2921, 2850, 1751, 1701, 1606, 1574, 1432, 1364, 1244, 1173, 1031. Elemental analysis (%) calculated for C_74_H_85_N_3_O_4_S_3_: C, 75.54; H, 7.28; N, 3.57; S, 8.17. Found: C, 72.97; H, 7.05; N, 3.38; S, 7.58.

## 3. Results and Discussion

### 3.1. Molecular Weights and Yield

The molecular weights of the polymers were measured by gel permeation chromatography (GPC) in chloroform solution at 40 °C relative to polystyrene standards (Table 1). Both polymers were synthesised in good yields and the number average molecular weight (*M*_n_) of toluene and chloroform fractions are comparable. The *M*_n_ for the toluene fractions of the polymers are relatively low. However, the *M*_n_ for the chloroform fractions of the polymers are almost twice than those of the toluene fractions. The results indicate that anchoring different chains (*n*-octyl vs. 3,7-dimethyloctyl) on the imide functionality of the BTDI building blocks provides polymers with similar processability and has a negligible impact on the *M*_n_ values of the resulting polymers.

### 3.2. Optical Properties

**Figure 3:**
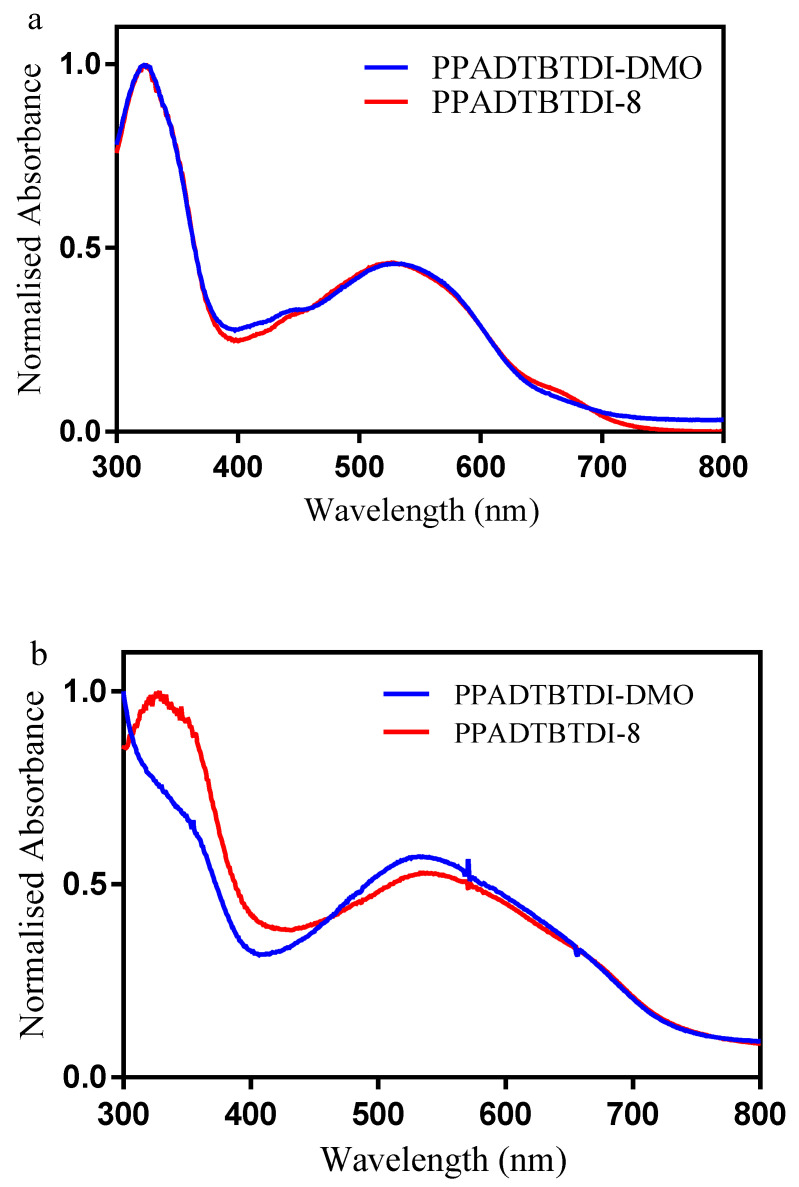
UV–vis absorption spectra of PPADTBTDI-DMO and PPADTBTDI-8 in (**a**) chloroform solutions; and (**b**) thin films.

The normalized UV–vis absorption spectra of the polymers in chloroform solutions and in thin-films are shown in Figure 3. The optical properties of these polymers are summarized in Table 2. PPADTBTDI-DMO and PPADTBTDI-8 display absorption maxima at 540 and 535 nm in solutions with shoulder absorption bands at 632 and 635 nm, respectively. The shoulder absorption bands may arise from π–π intermolecular interactions between the polymer chains and their aggregation in solutions. As compared to chloroform solutions, the absorption maxima in film states are slightly red shifted (5–11 nm) with stronger shoulder absorption bands at 660 nm for both PPADTBTDI-DMO and PPADTBTDI-8. This result indicates that both polymers show more pronounced aggregation in the solid state with the formation of more coplanar structures in thin films. The E_g_ of the polymers are assessed from the absorption onsets in thin films. Both polymers have comparable E_g_ values of 1.66 eV. The UV–vis absorption spectra revealed that attaching different chains on the imide functionality of the BTDI units does not have substantial influence on the optical properties of the polymers.

The absorption maxima and E_g_ of PPADTBTDI-DMO and PPADTBTDI-8 are comparable with PTATDPP and PTAFDPP analogues [23]. The E_g_ of PPADTBTDI-DMO and PPADTBTDI-8 are significantly lower than those of their PPATBT, PPATBT-8 and PPAT2BT-8 counterparts [21]. This may arise from the stronger electron withdrawing capability of BTDI units than BT and (OR)_2_BT moieties, consequently lowering the LUMO energy levels of BTDI-based polymers. The E_g_ of PPADTBTDI-DMO and PPADTBTDI-8 are significantly lower than that of fluorene-, dibenzosilole and carbazole-based polymers [38,39]. This is probably due to anthracene-based two-dimensional conjugated polymers have stronger and broader absorption bands [40]. The absorption coefficient (ε) of the PPADTBTDI-8 is significantly higher than PPADTBTDI-DMO despite the fact that they have comparable *M*_n_ values and E_g_ values.

### 3.3. Electrochemical Properties

**Figure 4:**
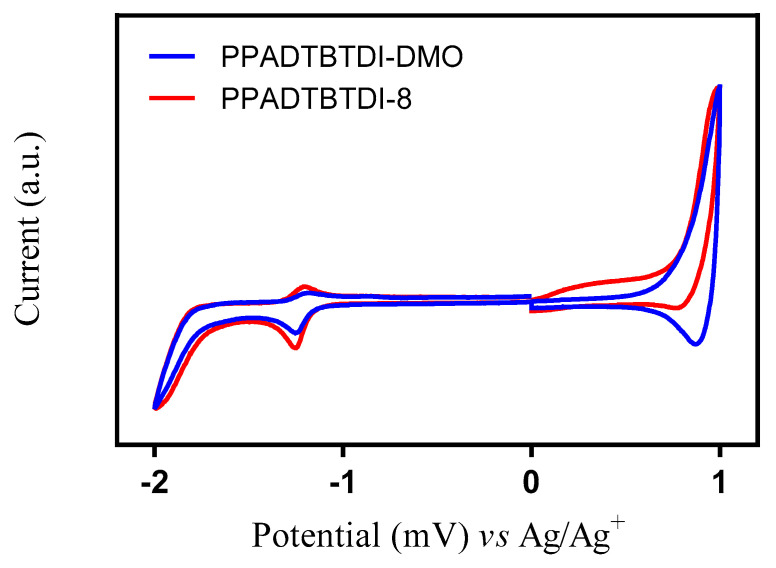
Cyclic voltammograms of PPADTBTDI-DMO and PPADTBTDI-8 on platinum disc electrodes (area 0.031 cm^2^) at a scan rate of 100 mV s^−1^ in acetonitrile/tetrabutylammonium perchlorate (0.1 mol dm^−3^).

Cyclic voltammetry was used to study the electrochemical properties of the polymers. The LUMO and HOMO levels of the polymers were calculated from the onsets of the reduction and oxidation potentials, respectively (Figure 4 and Table 3). The onsets were determined from cyclic voltammograms on drop cast polymer films on platinum electrode as working electrode in tetrabutylammonium perchlorate/acetonitrile (0.1 M) vs. Ag/Ag^+^ reference electrode. PPADTBTDI-DMO shows a reversible oxidation peak, while PPADTBTDI-8 displays an irreversible oxidation peak. Both polymers show one reversible reduction peak at higher potential and one irreversible reduction peak at the lower potential. The onset potentials of the first oxidation wave of PPADTBTDI-DMO and PPADTBTDI-8 appeared at 0.79 and 0.81 V vs. Ag/Ag^+^, corresponding to HOMO energy levels of −5.51 and −5.53 eV, respectively. The onset potential of the first reduction wave of PPADTBTDI-DMO and PPADTBTDI-8 appeared at −1.15 V, corresponding to a LUMO energy level of −3.56 eV. This corresponds to an electrochemical E_g_ of 1.95 and 1.97 eV for PPADTBTDI-DMO and PPADTBTDI-8, respectively. The HOMO levels of both polymers are comparable. The HOMO energy level is dominated by the nature of the donor unit and as both polymers have the same anthracene donor unit. Both polymers show deep-lying HOMO energy levels which could be beneficial for the chemical stability of the polymers in oxygen and might lead to higher *V*_oc_ values when fabricated in BHJ solar cells as donor materials with fullerene derivatives as acceptor materials. The LUMO levels of both polymers are similar, as both polymers have the same BTDI acceptor units which control the LUMO energy levels in these materials. Substituting the 3,7-dimethyloctyl chain with the *n*-octyl chain on the imide functionality of the BTDI units does not have a major effect on the electrochemical properties of the polymers.

The HOMO energy levels of PPADTBTDI-DMO and PPADTBTDI-8 are slightly lower than those of PPATBT and PPATBT-8, whereas they are significantly deeper than PPAT2BT-8 analogues. The shallower HOMO energy level of PPAT2BT-8 is mainly due to the flanking bithiophene units between the anthracene unit and (OR)_2_BT unit, which increase the intramolecular charge transfer along its polymer backbone. PPADTBTDI-DMO and PPADTBTDI-8 have very low-lying LUMO energy levels relative to those of their PPATBT, PPATBT-8 and PPAT2BT-8 counterparts [21]. This could be explained by the stronger electron-withdrawing ability of BTDI unit than BT and (OR)_2_BT moieties. These results could be promising as to the photovoltaic properties of these two polymers in BHJ solar cells with fullerene derivatives.

### 3.4. Thermal Properties

**Figure 5:**
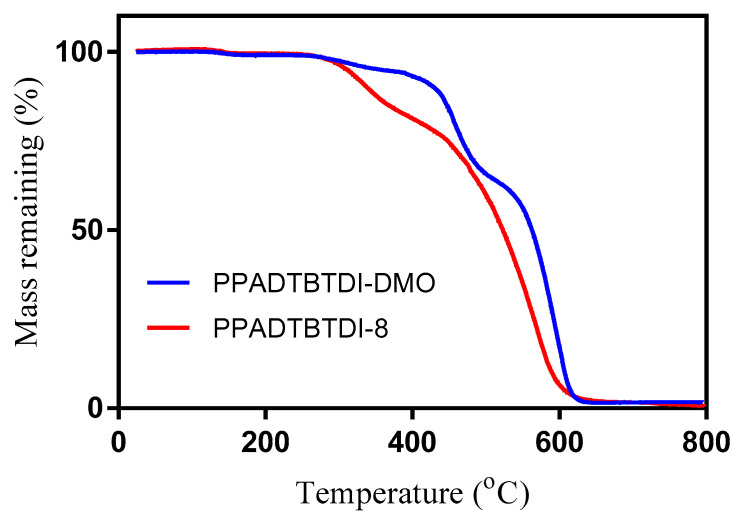
TGA of PPADTBTDI-DMO and PPADTBTDI-8.

The thermal properties of the polymers were studied by thermogravimetric analysis (TGA). TGA of PPADTBTDI-DMO and PPADTBTDI-8 did not detect changes below 354 and 313 ^°^C, respectively (Figure 5 and Table 3). The thermal stability of PPADTBTDI-8 is significantly lower than that of PPADTBTDI-DMO. It is worth noting that the thermal properties of the polymers essentially depend on the type of the alkyl chains was anchored to the imide functionality on the BTDI units. The polymers have thermal stability windows that are well within those used in solar cell applications and could be stable for such use. In previous studies, TGA has been used for evaluating the thermal properties of D–A conjugated polymers for polymer solar cell (PSC) applications [19,22,41,42,43,44].

### 3.5. Powder X-Ray Diffraction (XRD)

**Figure 6:**
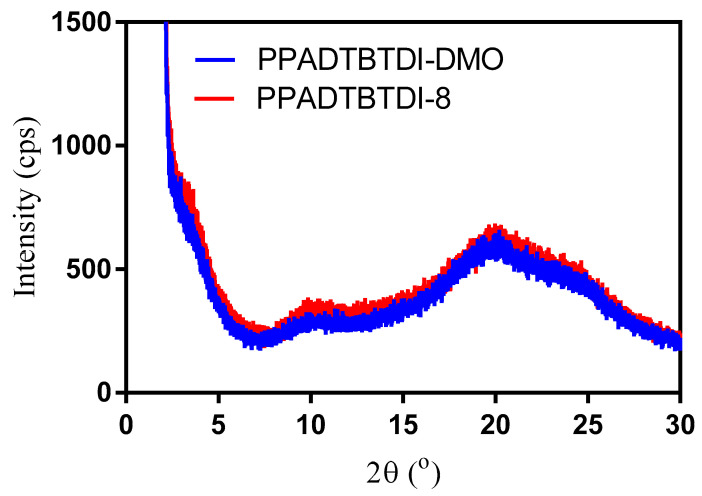
Powder XRD of PPADTBTDI-DMO and PPADTBTDI-8.

The structural properties of the polymers were studied by powder XRD in the solid state (Figure 6). PPADTBTDI-DMO and PPADTBTDI-8 exhibit a similar diffraction patent with a broad diffraction peak at an angle of 20.0°, corresponding to the π–π stacking distances between polymer chains of 0.443 nm. This result shows that both polymers have an amorphous nature which are similar to analogous anthracene-based polymers, PTATPD (O), PTATPD (DMO) and PTATPD (BP) [41]. Table 4 lists the symbols and their corresponding physical significances.

## 4. Conclusions

Two low band gap alternating copolymers comprising of 2,6-linked anthracene moieties flanked by thienyl units as electron donor units and benzothiadiazole dicarboxylic imide (BTDI) as electron acceptor units were synthesised through Suzuki polymerisation, and yielded PPADTBTDI-DMO and PPADTBTDI-8. Both polymers were prepared in good yields and show good solubility in common organic solvents. Both polymers have comparable *M*_n_ values for both toluene and chloroform fractions. The *M*_n_ values of the toluene fractions of the polymers are about 6000 g mol^−1^ which are relatively low. However, the *M*_n_ values of the chloroform fractions of the polymers are higher than 12,000 g mol^−1^. Both polymers show absorption maxima around 540 nm in chloroform solutions with shoulder absorption bands at about 635 nm. The shoulder absorption peak could be attributed to intermolecular interactions between the polymer chains and a certain degree of their aggregation in solutions. In thin films, the absorption spectra of the polymers show slightly bathochromic shift absorption maxima with a stronger shoulder at 660 nm relative to their absorption in solutions. This is related to those polymers adopting a more planar structure in thin-films. The E_g_ of the polymers are low (E_g_ = 1.66 eV) which could be beneficial to obtain high *J*_sc_ values in BHJ solar cells. The HOMO levels of both polymers are comparable because the HOMO energy level is controlled by the nature of the donor unit and both polymers have the same anthracene donor units. Both polymers show low-lying HOMO energy levels of about −5.5 eV which could be useful for the chemical stability of the polymers in oxygen and could lead to high *V*_oc_ values when fabricated in BHJ solar cells as donor materials with fullerene derivatives as acceptor materials. The LUMO energy levels of both polymers are similar, as both polymers have the same BTDI acceptor units which dominate the LUMO levels in these materials. Both polymers could show good thermal stability with T_d_ surpass 310 °C. Attaching different solubilizing chains (3,7-dimethyloctyl vs. *n*-octyl) on BTDI moieties has a little impact on the molecular weights and optoelectronic properties of the polymers. The TGA of PPADTBTDI-DMO and PPADTBTDI-8 did not prove instability with decomposition temperatures of 354 and 313 ^°^C, respectively. The powder XRD of the polymers showed diffraction peaks at 20.0° corresponding to the π–π stacking distance of 0.443 nm. Both polymers have the amorphous nature in the solid state, which could be employed as electrolytes in energy devices.

## Figures and Tables

**Table 1:** The percentage yield, number and weight average molecular weights with polydispersity indexes of PPADTBTDI-DMO and PPADTBTDI-8.

Polymer	% Yield	Toluene Fraction			Chloroform Fraction		
		*M*_n_ (g mol^−1^)	*M*_w_ (g mol^−1^)	Poly Dispersity Index (PDI)	*M*_n_ (g mol^−1^)	*M*_w_ (g mol^−1^)	PDI
PPADTBTDI-DMO	97	6700	12,600	1.8	12,700	22,400	1.7
PPADTBTDI-8	97	6000	11,000	1.8	12,500	27,400	2.1

**Table 2:** The UV–vis data and optical E_g_ of the polymers.

Polymer	ε (M^−1^ cm^−1^)	Solution	Film		
		λ_max_ (nm)	λ_max_ (nm)	λ_onset_ (nm)	E_g_ (eV)
PPADTBTDI-DMO	24,300	540	545	744	1.66
PPADTBTDI-8	34,000	535	546	744	1.66

**Table 3:** Thermal and electrochemical properties of the polymers.

Polymer	*T*_d_ (°C)	E_ox_^0^ (V)	HOMO (eV)	E_red_^0^ (V)	LUMO (eV)	E_g (elec)_ (eV)
PPADTBTDI-DMO	354	0.79	−5.51	−1.15	−3.56	1.95
PPADTBTDI-8	313	0.81	−5.53	−1.15	−3.56	1.97

**Table 4:** Symbols and their corresponding physical significances.

Symbols	Physical Significances
E_g_	Energy gap in eV
HOMO	Highest occupied molecular orbital level in eV
LUMO	Lowest unoccupied molecular orbital in eV
FET	Field effect transistor
BHJ	Bulk heterojunction
V_oc_	Open-circuit voltage in volts
J_sc_	Short-circuit current in ampere
PC	Power conversion
λ	Lambda in nm
T_d_	Degradation temperature in °C (5% weight loss)
D-A	Donor-acceptor
NBS	*N*-bromosuccinimide
DMF	*N*,*N*-dimethylformamide
DCM	Dichloromethane
BT	2,1,3-Benzothiadiazole
BTDI	Benzothiadiazole dicarboxylic imide
THF	Tetrahydrofuran
PPADTBTDI-DMO	Poly[9,10-bis(4-(dodecyloxy)phenyl)-2,6-anthracene-alt-5,5-(4′,7′-bis(2-thienyl)-2′,1′,3′-benzothiadiazole-*N*-5,6-(3,7-dimethyloctyl)dicarboxylic imide)]
PPADTBTDI-8	Poly[9,10-bis(4-(dodecyloxy)phenyl)-2,6-anthracene-alt-5,5-(4′,7′-bis(2-thienyl)-2′,1′,3′-benzothiadiazole-5,6-*N*-octyl-dicarboxylic imide)]
XRD	Powder X-ray diffraction
PSC	Polymer solar cell
PDPP-FAF	Poly[3,6-difuran-2-yl-2,5-di(2-octyldodecyl)-pyrrolo[3,4-*c*]pyrrole-1,4-dione-*alt*-anthracene]
PPATBT	Poly(9,10-bis(4-(dodecyloxy)phenyl)-anthracene-2,6-diyl-alt-(4,7-dithiophen-2-yl)-2′,1′,3′-benzothiadiazole-5,5-diyl]
PPATBT-8	Poly(9,10-bis(4-(dodecyloxy)phenyl)-anthracene-2,6-diyl-alt-(5,6-bis(octyloxy)-4,7-di(thiophen-2-yl)benzo[c][1,2,5]thiadiazole-5,5-diyl]
PPAT2BT-8	Poly(9,10-bis(4-(dodecyloxy)phenyl)-anthracene-2,6-diyl-alt-(5,6-bis(octyloxy)-4,7-di(2,2′-bithiophen-5-yl)benzo[c][1,2,5] thiadiazole)-5,5-diyl]
PTATffBT	Poly[9,10-bis(triisopropylsilylacetylene)-anthracene-2,6-diyl-alt-(4,7-dithiophen-2-yl)-5,6-difluorobenzo[c][1,2,5]thiadiazole-5-5-diyl]
PhNSO	*N*-thionyl aniline
TMSCl	Trimethylsilyl chloride
M1	4,7-Di(5-bromo-thien-2-yl)-2,1,3-benzothiadiazole-5,6-*N*-(3,7-dimethyloctyl)dicarboxylic imide
M2	4,7-Di(5-bromo-thien-2-yl)-2,1,3-benzothiadiazole-5,6-*N*-octyl-dicarboxylic imide
GPC	Gel permeation chromatography
PTATBT-8	Poly[9,10-bis(triisopropylsilylacetylene)-anthracene-2,6-diylalt-(5,6-bis(octyloxy)-4,7-di(thiophene-2-yl)benzo[c]thiadiazole-5,5-diyl]
